# Recombinant *Lactococcus lactis* expressing porcine insulin-like growth factor I ameliorates DSS-induced colitis in mice

**DOI:** 10.1186/s12896-016-0255-z

**Published:** 2016-03-01

**Authors:** Shujie Liu, Yongming Li, Bo Deng, Ziwei Xu

**Affiliations:** Institute of Animal Husbandry and Veterinary Science, Zhejiang Academy of Agricultural Sciences, 145 Shiqiao Road, Hangzhou, 310021 Zhejiang China

**Keywords:** Insulin-like growth factor I, *Lactococcus lactis*, Colitis, Intestinal repair

## Abstract

**Background:**

Insulin-like growth factor I (IGF-I) is one important family of growth factors, which plays key role in intestinal growth, regeneration, and damage repair. However, the low natural abundance of IGF-I limits its research opportunities and practical application in the fields of medicine and animal husbandry. In this study, a tandem repeat strategy was used to express three copies of the same pIGF-I_3_ protein in *L. lactis*. The activity of recombinant pIGF-I_3_ (rpIGF-I_3_) was further examined by a mouse model of dextran sulfate sodium (DSS)–induced colitis. In addition, the potential of recombinant *L. lactis* expressing pIGF-I_3_ to reduce inflammatory disease was evaluated.

**Results:**

pIGF-I_3_ could be expressed in *L. lactis* by the detection of SDS–PAGE and Western blot. Experimental colitis was induced in BALB/c mice by administration of 5 % DSS in drinking water, and the clinical symptoms were observed in DSS-treated mice. Oral administration of recombinant *L. lactis* expressing pIGF-I_3_ improved the colonic architecture, and significantly reduced the increase of colonic damage score (*P* < 0.05). Furthermore, recombinant *L. lactis* expressing pIGF-I_3_ treatment significantly reduced serum DAO activity and colonic MPO level, and elevated colonic occludin level compared to the DSS group (*P* < 0.05).

**Conclusions:**

The pIGF-I_3_ expressed in *L. lactis* has good biological activity, and oral administration of recombinant *L. lactis* expressing pIGF-I_3_ attenuated the symptoms and development of DSS-induced colitis in mice. These suggested that *L. lactis* could be a potential host bacterium for production and delivery of IGF-I against intestinal diseases.

## Background

Insulin-like growth factor I (IGF-I) is a 7.5 kDa single-chain polypeptide with 70 amino acid residues, which is considered as one important family of growth factors [1]. IGF-I is synthesized in the liver, produced locally in many tissues including the gastrointestinal tract, and regulated by growth hormone and nutrition [2, 3]. It exerts biological effect primarily through specific type I IGF receptor, which is distributed throughout the intestines of some mammalian, such as human, rat and pig [4, 5]. IGF-I can stimulate the proliferation and differentiation of many cell types, enhance DNA synthesis and protein content, inhibit cell apoptosis, and play an important role in the development and growth of animals [6–11]. In addition, IGF-I particularly displays trophic effect on the gastrointestinal tract, which has received considerable attention in the fields of medicine and animal husbandry.

Studies have indicated that IGF-I not only stimulated intestinal growth but also potentially regulated intestinal regeneration and repair. High concentrations of IGF-I in maternal colostrum and milk modulated neonatal gastrointestinal tract development and function [12]. IGF-I supplementation in newborn promoted nutrient and electrolyte absorption, improved intestinal morphology, and increased disaccharidase activity [13, 14]. In addition, orally or systemically administered IGF-I accelerated intestinal repair in animal models of intestinal diseases, such as experimental colitis, intestinal mucositis, and experimental radiation enteritis [15–17]. IGF-I has showed the potential to exert anti-inflammatory action by inhibiting pro-inflammatory cytokine production and increasing anti-inflammatory cytokine production in acute pancreatitis [18].

IGF-I has important biological functions, but low abundance in natural sources is a crucial factor to limit its further study and practical application. Genetic engineering technique is a good method for low-cost production of functional proteins by large-scale culture of recombinant bacteria. Therefore, a safe and highly effective expression system is needed to produce functional proteins for the health of humans and animals. Human IGF-I has been produced in *E. coli*, but recombinant protein was expressed in insoluble inclusion bodies, and needed to be refolded into an active conformation by denaturation and renaturation treatment [19]. Probiotics has been reported to improve host health and modulate the gastrointestinal functions in human and animals [20]. *Lactococcus lactis* (*L. lactis*) is a food-grade microorganism widely used in the food fermentation industry and generally regarded as a safe probiotic [21]. Nonpathogenic and noninvasive *L. lactis* is an ideal candidate to produce and deliver therapeutic proteins to mucosal system of intestine with simple operation [22]. Furthermore, the bacterium can survive passage through human intestines and does not colonize the intestinal tract [23]. Foreign proteins expressed in *L. lactis* do not require to be purified, may be taken together with recombinant *L. lactis*, and can perform specific biological activity in body [24].

In the present study, three same genes of mature porcine IGF-I (pIGF-I_3_) were designed by tandem repeat strategy, and a nisin-controlled gene expression system (NICE) was used to express pIGF-I_3_ in *L. lactis*. The activity of recombinant pIGF-I_3_ (rpIGF-I_3_) was examined by using a mouse model of dextran sulfate sodium (DSS)–induced colitis, and the potential of recombinant *L. lactis* expressing pIGF-I_3_ to reduce inflammatory disease was evaluated.

## Results and discussion

### Expression of pIGF-I_3_ in *L. lactis*

**Fig. 1:**
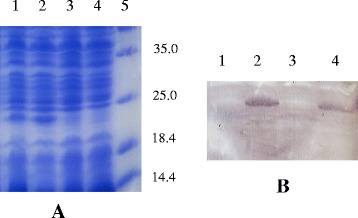
Detection of rpIGF-I_3_ by SDS–PAGE (**a**) and Western blot (**b**) analysis of cell lysates of *L. lactis* NZ9000 (pNZ8148-pIGF-I_3_) induced with nisin. **a** Lanes 1 and 2, cell lysates of *L. lactis* NZ9000 (pNZ8148-pIGF-I_3_); lanes 3 and 4, cell lysates of *L. lactis* NZ9000 (pNZ8148); lane 5, molecular weight marker. **b** Lanes 1 and 3, cell lysates of *L. lactis* NZ9000 (pNZ8148); lanes 2 and 4, cell lysates of *L. lactis* NZ9000 (pNZ8148-pIGF-I_3_)

The gene pIGF-I_3_ was optimized based on codon bias of *L. lactis*, and the restriction sites and a stop codon were added to the 3′ or 5′ end of the optimized pIGF-I_3_ gene. The resulting fragment was cloned into pNZ8148 to generate pNZ8148-pIGF-I_3_, which had been successfully transformed into *L. lactis* NZ9000 by sequence identification. Whole cell lysates of *L. lactis* NZ9000 (pNZ8148-pIGF-I_3_) were analyzed by using sodium dodecyl sulfate–polyacrylamide gel electrophoresis (SDS–PAGE) to determine whether pIGF-I_3_ could be expressed in *L. lactis* (Fig. 1a). The protein electrophoresis of the gel revealed an additional band of approximately 23 kDa, which just corresponded to the target protein of pIGF-I_3_. However, *L. lactis* NZ9000 (pNZ8148) as control strain did not have this band. The rpIGF-I_3_ was further assayed by Western blot, and the result revealed that the corresponding immunoreactive band was present on the membrane from *L. lactis* NZ9000 (pNZ8148-pIGF-I_3_) (Fig. 1b). These results indicated that *L. lactis* was able to express pIGF-I_3_.

### Colon length analysis

**Fig. 2:**
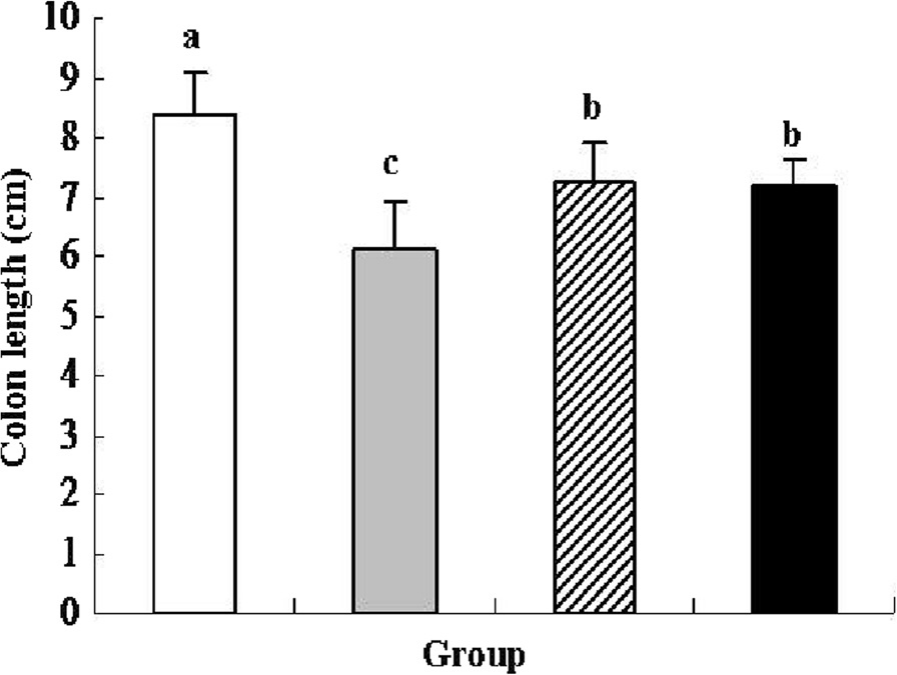
Effect of oral administration of *L. lactis* NZ9000 (pNZ8148-pIGF-I_3_) on colon length in mice (*n* = 8) treated with DSS-induced colitis. The statistical differences between groups were evaluated by one-way ANOVA with Duncan’s multiple comparison. Group 1 (control ) received normal drinking water. Group 2 (DSS ) received DSS solution in drinking water. Group 3 (*L. lactis* ) received the same treatment of DSS as Group 2. Meanwhile, these mice orally administered with *L. lactis* NZ9000 (pNZ8148). Group 4 (recombinant *L. lactis* ) received the same treatment of DSS as Group 2. Meanwhile, these mice orally administered with *L. lactis* NZ9000 (pNZ8148- pIGF-I_3_). Same letters between the groups indicate no statistically significant difference (P > 0.05), and different letters indicate statistically significant difference (*P* < 0.05)

The reduction of colon length was an indirect marker of colonic inflammation and often observed in ulcerative colitis patients or animal models [25]. Treatment with 5 % DSS significantly shortened colon length (31.67 %) compared with the control group (*P* < 0.05). However, oral administration of recombinant *L. lactis* expressing pIGF-I_3_ or *L. lactis* improved the shortening of colon length (Fig. 2). Dietary colostrum-borne IGF-I during one week has been reported to promote the growth of neonatal intestinal tissue and up-regulated type I IGF receptors in newborn calves [26]. In addition, IGF-I treatment partially reversed significant reduction of colon length in DSS-treated rats, and reduced the thickness of submucosal and muscularis externa layers [27]. In the present study, oral administration of recombinant *L. lactis* expressing pIGF-I_3_ significantly increased colon length by approximately 18.03 % compared with the DSS group (*P* < 0.05), but no significant difference was found between the recombinant *L. lactis* and *L. lactis* groups. These suggested that recombinant *L. lactis* expressing pIGF-I_3_ attenuated the shortening of colon length due to the use of *L. lactis* as a host bacterium, and took a certain protective effect on intestinal injury.

### Colonic damage score and histological analysis

**Fig. 3:**
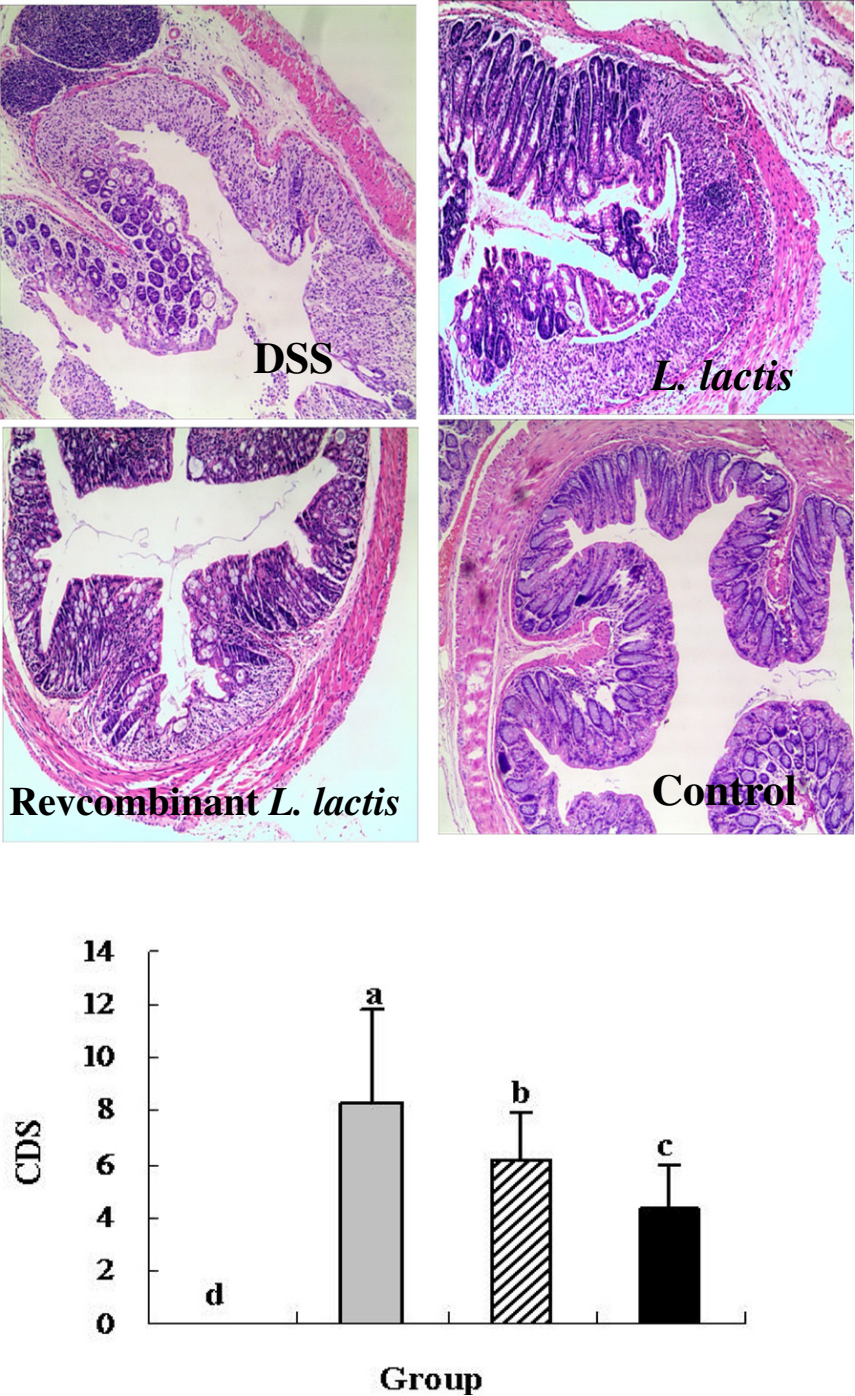
Effect of oral administration of *L. lactis* NZ9000 (pNZ8148-pIGF-I_3_) on histopathological changes and colonic damage score in mice (*n* = 6) treated with DSS-induced colitis. The statistical differences between groups were evaluated by one-way ANOVA with Duncan’s multiple comparison. Group 1 (control ) received normal drinking water. Group 2 (DSS ) received DSS solution in drinking water. Group 3 (*L. lactis* ) received the same treatment of DSS as Group 2. Meanwhile, these mice orally administered with *L. lactis* NZ9000 (pNZ8148). Group 4 (recombinant *L. lactis* ) received the same treatment of DSS as Group 2. Meanwhile, these mice orally administered with *L. lactis* NZ9000 (pNZ8148-pIGF-I_3_). Same letters between the groups indicate no statistically significant difference (P > 0.05), and different letters indicate statistically significant difference (*P* < 0.05)

Colonic damage was observed on the 11th day of the experiment by histological detection (Fig. 3). DSS treatment revealed a typical ulcer that involved most of intestine mucosa, seriously affected colonic architecture, and led to a significant increase in colonic damage score (CDS) compared with the control group (*P* < 0.05). As vector control, *L. lactis* strain treatment showed visible large ulcer surface, peripheral epithelial cells crawling to the ulcer surface, damage being repaired in the colonic tissue and a significant reduction CDS compared with the DSS group (*P* < 0.05). In the recombinant *L. lactis* group, the morphological structure of colonic tissue became relatively complete, and colon displayed visible small ulcer surface. In addition, oral administration of recombinant *L. lactis* expressing pIGF-I_3_ further improved colonic damage, and significantly reduced CDS compared with the DSS and *L. lactis* groups (*P* < 0.05). These results suggested a direct action of recombinant *L. lactis* expressing pIGF-I_3_ on the colonic epithelium.

It has been reported that exogenously applied IGF-I increased the numbers of goblet cells of the colonic epithelium, and significantly increased histological healing scores in an experimental model of colitis [27, 28]. Administration of IGF-I to cirrhotic rats improved intestinal histopathological changes and significantly reduced endotoxaemia [29]. Furthermore, IGF-I treatment restored gut weight to control levels and increased the number of crypt mitoses in rats with colon anastomoses [30]. In the present study, oral administration of recombinant *L. lactis* expressing pIGF-I_3_ reduced intestinal damage, and maintained the relative integrity of intestinal tract in DSS-treated mice.

Although oral administration of recombinant *L. lactis* expressing pIGF-I_3_ significantly increased colon length and reduced CDS compared with the DSS group, the colon length and CDS was only partially improved. This may perhaps be associated with the choice of animal models. In the present study, an acute DSS model of colitis is induced by continuous administration of 5 % DSS for 7 consecutive days. However, susceptibility of mice to DSS was affected by the concentration and molecular weight of DSS, genetic and microbiological factors of animals [31]. These were likely to influence the results of recombinant *L. lactis* expressing pIGF-I_3_ treatment. Genetic variations were well known to be important in intestinal dysfunction, such as IBD. IL-10-deficient (IL-10−/−) mice, a gene-targeted mutation, spontaneously develop a chronic enterocolitis due to a dysregulated immune response to ordinary enteric antigens [32]. This model might be more suitable to evaluate biological activity of the rpIGF-I_3_.

### Colonic myeloperoxidase activity analysis

**Fig. 4:**
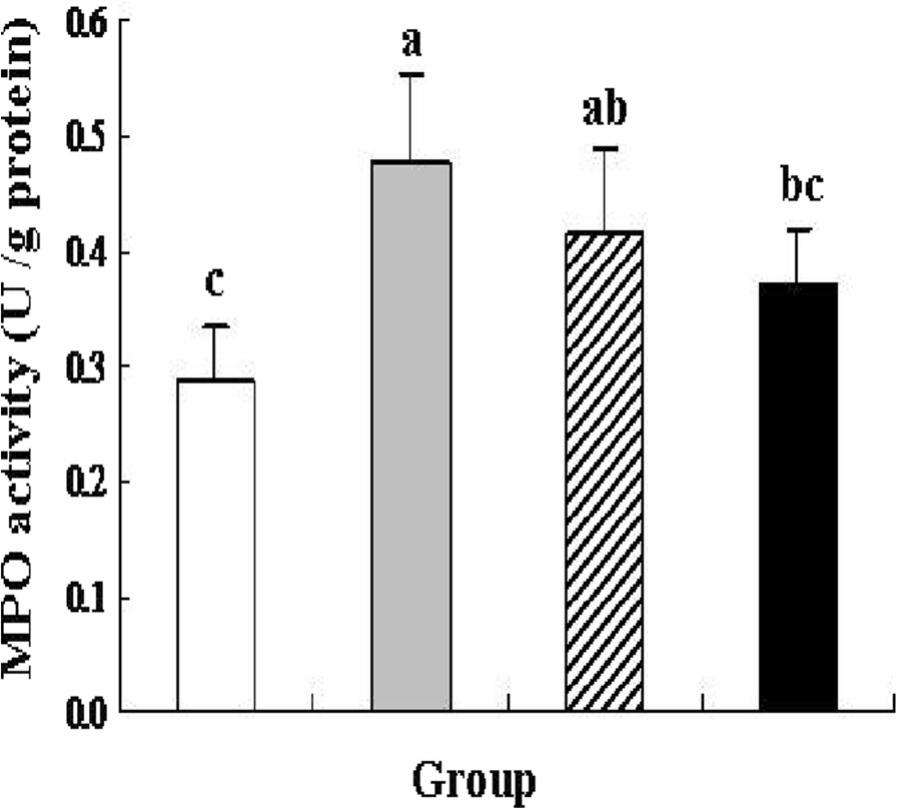
Effect of *L. lactis* NZ9000 (pNZ8148-pIGF-I_3_) on colonic MPO activity in mice (*n* = 6) treated with DSS-induced colitis. The statistical differences between groups were evaluated by one-way ANOVA with Duncan’s multiple comparison. Group 1 (control ) received normal drinking water. Group 2 (DSS ) received DSS solution in drinking water. Group 3 (*L. lactis* ) received the same treatment of DSS as Group 2. Meanwhile, these mice orally administered with *L. lactis* NZ9000 (pNZ8148). Group 4 (recombinant *L. lactis* ) received the same treatment of DSS as Group 2. Meanwhile, these mice orally administered with *L. lactis* NZ9000 (pNZ8148-pIGF-I_3_). Same letters between the groups indicate no statistically significant difference (P > 0.05), and different letters indicate statistically significant difference (*P* < 0.05)

Myeloperoxidase (MPO) is an enzyme produced in leukocytes, and its activity is linearly related to neutrophil infiltration of the colon as an index of inflammatory response under pathological conditions [33, 34]. In the present study, DSS treatment significantly increased MPO activity by approximately 65.51 % compared with the control group (*P* < 0.05), and the *L. lactis* group revealed a reduction trend on MPO activity (Fig. 4). Oral administration of recombinant *L. lactis* expressing pIGF-I_3_ significantly reduced MPO activity by approximately 20.83 % compared with the DSS group (*P* < 0.05), and showed no significant difference compared with the control group. A similar result was shown that IGF-I inhibited the increases of gastric MPO activity and immunofluorescence intensity of MPO in the gastric mucosa, and reduced stress-induced mucosal injury in mice [35]. The results of this study suggested that recombinant *L. lactis* expressing pIGF-I_3_ has an anti-inflammatory effect, might reduce intestinal mucosa injury partly because of inhibiting the accumulation of neutrophils in the colonic mucosa. In addition, *L. lactis* as a host bacterium contributed to beneficial effect on recombinant *L. lactis* expressing pIGF-I_3_ in the prevention of intestinal inflammation.

### Diamine oxidase activity and occludin level analysis

**Fig. 5:**
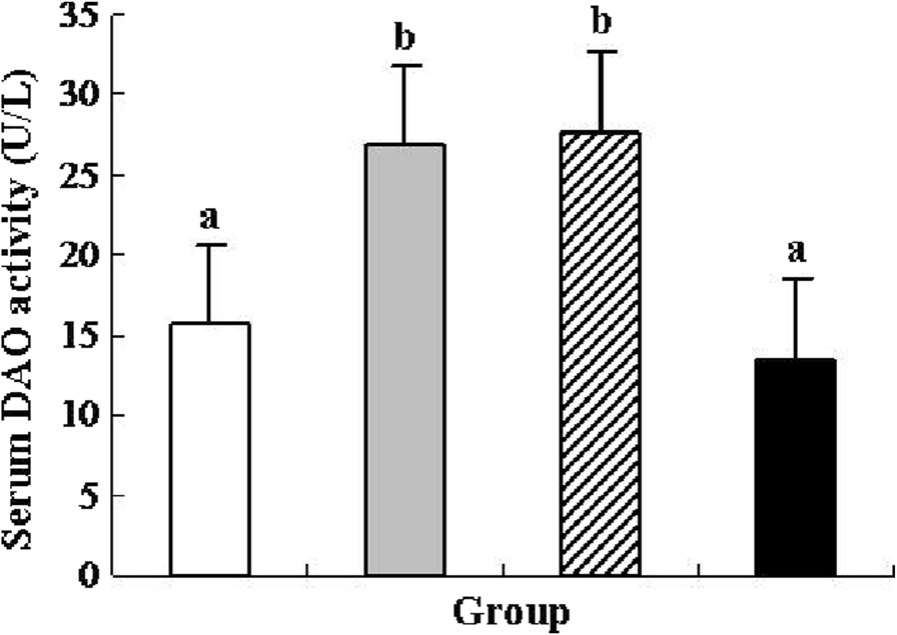
Effect of *L. lactis* NZ9000 (pNZ8148-pIGF-I_3_) on serum DAO activity in mice (*n* = 6) treated with DSS-induced colitis. The statistical differences between groups were evaluated by one-way ANOVA with Duncan’s multiple comparison. Group 1 (control ) received normal drinking water. Group 2 (DSS ) received DSS solution in drinking water. Group 3 (*L. lactis* ) received the same treatment of DSS as Group 2. Meanwhile, these mice orally administered with *L. lactis* NZ9000 (pNZ8148). Group 4 (recombinant *L. lactis* ) received the same treatment of DSS as Group 2. Meanwhile, these mice orally administered with *L. lactis* NZ9000 (pNZ8148-pIGF-I_3_). Same letters between the groups indicate no statistically significant difference (*P* > 0.05), and different letters indicate statistically significant difference (*P* < 0.05)

Diamine oxidase (DAO) is a cytoplasmic enzyme existing in the villus cytoplasm of intestinal stratum supravasculare. It is released and enters into the bloodstream when the intestinal mucosa is damaged. Therefore, serum DAO activity is often used as a marker of intestinal mucosal integrity [36]. In this study, DSS treatment significantly increased serum DAO activity by approximately 71.21 % compared with the control group (*P* < 0.05), and oral administration of *L. lactis* did not inhibit the increase of DAO activity (Fig. 5). However, oral administration of recombinant *L. lactis* expressing pIGF-I_3_ significantly reduced DAO activity by approximately 49.68 % compared with the DSS group (*P* < 0.05). No significant difference was found between the recombinant *L. lactis* and control groups.

**Fig. 6:**
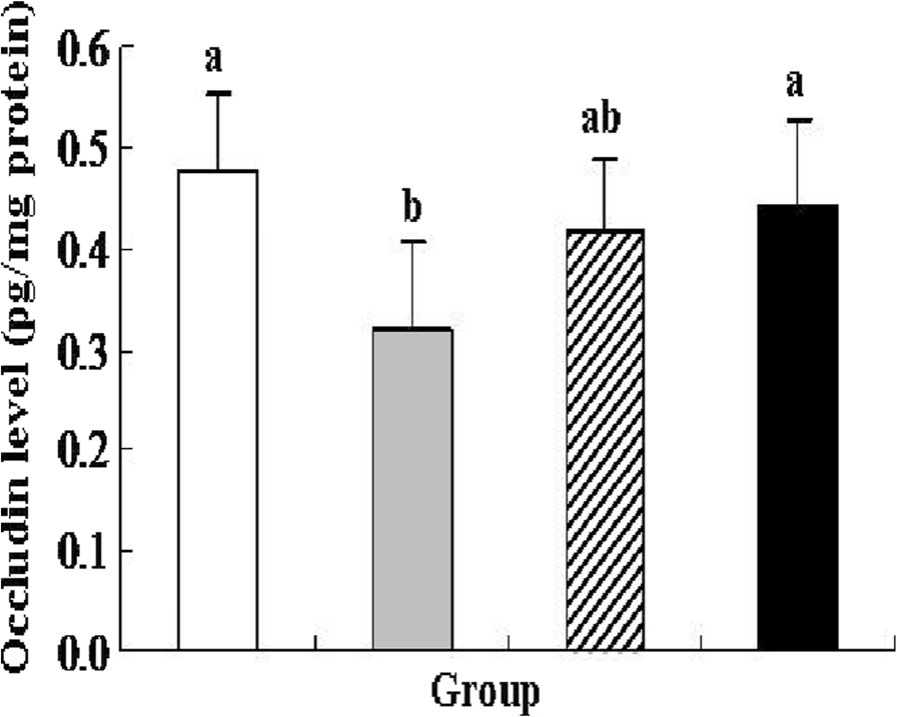
Effect of *L. lactis* NZ9000 (pNZ8148-pIGF-I_3_) on colonic occludin level in mice (*n* = 6) treated with DSS-induced colitis. The statistical differences between groups were evaluated by one-way ANOVA with Duncan’s multiple comparison. Group 1 (control ) received normal drinking water. Group 2 (DSS ) received DSS solution in drinking water. Group 3 (*L. lactis* ) received the same treatment of DSS as Group 2. Meanwhile, these mice orally administered with *L. lactis* NZ9000 (pNZ8148). Group 4 (recombinant *L. lactis* ) received the same treatment of DSS as Group 2. Meanwhile, these mice orally administered with *L. lactis* NZ9000 (pNZ8148-pIGF-I_3_). Same letters between the groups indicate no statistically significant difference (P > 0.05), and different letters indicate statistically significant difference (*P* < 0.05)

Tight junction (TJ) is the most important structure of intestinal barrier. It maintains cell polarity and regulates the permeability of ions, macromolecules, and cells through the paracellular pathway [37]. Occludin is an integral membrane protein localizing at TJ, and performs important functions in TJ assembly and maintenance [38]. Colonic occludin concentration was also measured in colon tissue. DSS treatment significantly decreased occludin level by approximately 33.53 % compared with the control group (*P* < 0.05) (Fig. 6). As vector control, *L. lactis* strain treatment enhanced occludin level compared with the DSS group, but no significant difference was found between the two groups. Oral administration of recombinant *L. lactis* expressing pIGF-I_3_ significantly increased occludin level by approximately 31.25 % compared with the DSS group (*P* < 0.05). No significant difference was found between the recombinant *L. lactis* and control groups.

IGF-I has been shown to maintain a paracellular barrier function in cells by the expression and distribution of TJ proteins and inhibit the increase of plasma DAO activity [39, 40]. In the present study, oral administration of recombinant *L. lactis* expressing pIGF-I_3_ prevented the increase of serum DAO activity, enhanced colonic occludin level in mice with colitis, and regulated their levels near to normal concentration. The results showed the consistency with histological examinations. These suggested that oral administration of recombinant *L. lactis* expressing pIGF-I_3_ protected the intestinal barrier and integrity, and decreased colitis-induced elevated epithelial permeability.

## Conclusions

In this study, we constructed recombinant *L. lactis* expressing pIGF-I_3_ and investigated its effect on intestinal regeneration and repair by using a mouse model of DSS-induced colitis. The results indicated that rpIGF-I_3_ has good biological activity. Oral administration of recombinant *L. lactis* expressing pIGF-I_3_ attenuated DSS-induced colitis and protected intestinal function by improving CDS, MPO and DAO activities, and inhibiting the increase of colonic occludin level. These suggested that IGF-I expressed in *L. lactis* could be a good way to treat intestinal diseases.

## Methods

### Bacterial strains, plasmids, and growth conditions

*L. lactis* NZ9000 and plasmid pNZ8148 of the NICE system were purchased from NIZO Food Research. *L. lactis* was grown as a host bacterium in an M17 medium (Difco, Sparks, Maryland, USA) supplemented with 0.5 % (w/v) glucose at 30 °C without agitation. *Escherichia coli* MC1061 was used as an intermediate cloning host and grown in Luria–Bertani medium with agitation at 37 °C. Chloramphenicol was used at a concentration of 10 μg/mL to select recombinant strains.

### Optimization of codon and construction of recombinant *L. lactis*

pIGF-I is a small protein composed of 70 amino acid residues with a molecular weight of 7.5 kDa. In order to prevent pIGF-I degradation by cellular enzyme, three copies of mature pIGF-I (pIGF-I_3_) with the same nucleotide sequence were designed by tandem repeat strategy according to pIGF-I mRNA (GenBank accession no. M31175.1), and separated by a 18 bp linker that yields the amino acid sequence GGGGSG between the copies of pIGF-I. The gene sequence of the designed pIGF-I_3_ was further optimized based on codon bias of *L. lactis* (http://www.kazusa.or.jp/codon), and low-usage codons were replaced by high-usage ones. *Nco*I restriction site (CCATGG) containing a start codon (ATG) was added to the 5′ end of the first copy, a stop codon (TAA) and *Hind*III restriction site (AAGCTT) were added to the 3′ end of the third copy. Furthermore, the G + C content was adjusted to 40 to 70 %, and the resulting fragment (665 bp) was synthesized by Shanghai Shengong Biotechonology (Shanghai, China).

The resulting fragment was cloned into *Nco*I and *Hin*dIII sites of pNZ8148 vector, and this recombinant vector was transformed into *E. coli* MC1061. The colonies were selected by resistance to chloramphenicol, confirmed by digestion with *Nco*I and *Hind*III, and further sequenced by Invitrogen (Shanghai, China). The positive plasmid named pNZ8148-pIGF-I_3_ was then transformed into *L. lactis* NZ9000 by electroporation.

### Expression and identification of rpIGF-I_3_ in *L. lactis*

The recombinant protein of pIGF-I_3_ was identified by SDS–PAGE and Western blot as previously described [41]. Briefly, *L. lactis* NZ9000 (pNZ8148-pIGF-I_3_) and *L. lactis* NZ9000 (pNZ8148) were inoculated (3 % v/v) in fresh medium and grown to an optical density at OD_600_ = 0.4. Then these strains were induced with 10 ng/mL of nisin (Sigma-Aldrich, USA) and continued to grow for 3 h. After harvested by centrifugation, the strains were suspended in 10 mM Tris–HCl buffer (pH 8.0) containing 1 mg/mL lysozyme and incubated on ice for 30 min. The strain extracts were obtained by centrifugation for 10 min, and 10 μL of aliquots was analyzed by using 10 % SDS–PAGE. Subsequently, the gel with proteins was transferred to polyvinylidene fluoride membrane (Millipore, Bedford, MA, USA) using a semidry blotting apparatus (Bio-Rad, Hercules, CA, USA). The membrane was incubated with primary anti-IGF-I monoclonal antibody (Abcam, Cambridge, MA, USA) and HRP-conjugated goat polyclonal secondary antibody (Abcam), followed by 3,3′-diaminobenzidine as the substrate for visualizing bands.

### Stains preparation for animal experiment

*L. lactis* NZ9000 (pNZ8148-pIGF-I_3_) and *L. lactis* NZ9000 (pNZ8148) were grown and induced as described above. Stains were collected and washed three times with 0.01 mol/L sterile phosphate-buffered saline (PBS) after centrifugation. Finally, *L. lactis* NZ9000 (pNZ8148-pIGF-I_3_) and *L. lactis* NZ9000 (pNZ8148) were suspended in PBS to a concentration of 4 × 10^12^ CFU, respectively.

### Animals and DSS-induced colitis

Eight-week-old female BALB/c mice were purchased from SLAC Laboratory Animal Central (Shanghai, China). These pathogen-free mice were housed in filter-top cages at experimental animal room under standard condition, and were provided with ad libitum water and food. All animal protocols were carried out in strict accordance with the guidelines of the Animal Care and Use Committee of Zhejiang, China, and approved by the Committee on the Ethics of Animal Experiments of Zhejiang Academy of Agricultural Sciences, China.

Animals were randomly allotted in five groups, eight mice per group. Mice were treated at 6 weeks of age. Group 1 (control group) received normal drinking water for 10 days. Group 2 (DSS group) received 5 % w/v DSS solution (MW 36,000–50,000; MP Biomedicals, LLC, Illkirch, France) in drinking water for 7 consecutive days and followed with normal drinking water for 3 days. Group 3 (*L. lactis* group) received the same treatment of DSS as Group 2. Meanwhile, these mice were orally administered with 4 × 10^12^ CFU of *L. lactis* NZ9000 (pNZ8148) for 10 days. Group 4 (recombinant *L. lactis* group) received the same treatment of DSS as Group 2. Meanwhile, these mice were orally administered with 4 × 10^12^ CFU of *L. lactis* NZ9000 (pNZ8148-pIGF-I_3_) for 10 days.

All mice were anesthetized with pentobarbital sodium and killed on the 11th day of the experiment. The colons of all mice were removed, and the length was measured. In addition, colon and blood samples of six mice in each group were randomly selected for the following analysis. Samples of the distal colon approximately 1 cm were immersion-fixed for 24 h in 10 % v/v formalin buffer for histological assay. Other colonic segments were frozen at −80 °C to analyze MPO activity, and occludin level. Before they were killed, blood samples of mice were collected by orbital puncture on the 11th day of the experiment. Serum was obtained for analysis of DAO activity by centrifugation at 1500 × *g* for 15 min at 4 °C.

### Histology analysis for colonic damage score

Tissue samples of colon were embedded in paraffin following standard histology procedures. Subsequently, the blocks were cut into sections of 4 μm thickness, which were stained routinely with hematoxylin and eosin, and microscopically examined. Colonic damage was graded in a blinded manner as described by Dieleman et al. [42]. The colonic sections were briefly assessed based on the amount of inflammation (acute and chronic; scored from 0 to 3), the depth of inflammation, and the amount of crypt damage or regeneration (scored from 0 to 4). The results were quantified for the percentage involvement in the disease process as follows: (1) 1 to 25 %, (2) 26 to 50 %, (3) 51 to 75 %, and (4) 76 to 100 %. The score of each colonic section was provided for each feature by establishing the product of the grade for that feature and the percentage involvement. Finally, the colonic damage score (CDS) of each mice was the sum of these scores.

### Determination of colonic myeloperoxidase activity and occludin level

MPO activity was measured in colon tissue using the Assay Kit following the manufacturer’s instruction (Nanjing Jiancheng Bio-engineering Co., Ltd., China). One unit of MPO activity was defined as H_2_O_2_ broken into 1 mol in each gram of tissue at 37 °C, and the result was finally expressed as units per gram of total colon protein. The occludin level of colon tissue was analyzed using the Mouse ELISA kit (Nanjing Jiancheng Bio-engineering Co., Ltd.) and expressed as nanograms per milligram of total colon protein.

### Measurement of serum diamine oxidase activity

The activity of serum DAO was further measured using the Assay Kit following the manufacturer’s instruction (Nanjing Jiancheng Bio-engineering Co., Ltd.). DAO activity was expressed as units per liter of serum volume.

### Statistical analysis

All data were presented as means ± standard deviation for each group. The statistical differences between groups were evaluated by one-way ANOVA with Duncan’s multiple comparison. *P* < 0.05 was considered statistically significant.

## Abbreviations

CDS: Colonic damage score
DAO: Diamine oxidase
DSS: Dextran sulfate sodium
IGF-I: Insulin-like growth factor I
*L. lactis*: *Lactococcus lactis*
MPO: Myeloperoxidase
PBS: Phosphate-buffered saline
pIGF-I: Porcine insulin-like growth factor I
pIGF-I_3_: Three same genes of mature porcine IGF-I
rpIGF-I_3_: Recombinant pIGF-I_3_
SDS–PAGE: Sodium dodecyl sulfate–polyacrylamide gel electrophoresis
